# Supplementary material for: Are carbon emissions trading and green financial instruments synergistic? -Comprehensive quantitative research based on content analysis
Source: PLoS One. 2024 Mar 7;19(3):e0298601. doi: 10.1371/journal.pone.0298601 (PMC10919601; doi:10.1371/journal.pone.0298601)
Supplement: S1 Data — (ZIP) [file pone.0298601.s001.zip › Data/地级市Z_F工作报告.zip!/╕╩╦α/╬Σ═■╩╨/└┤╫╘╣1⁄2╓┌║┼í╛┬φ┐╦╩2╛▌═°í┐_2016.docx]

各位代表：

现在，我代表市人民政府向大会作工作报告，请予审议，并请政协委员和列席人员提出意见。

“十二五”及2015年工作回顾

“十二五”时期，面对复杂多变的经济形势和艰巨繁重的改革发展稳定任务，在省委、省政府和市委的坚强领导下，全市上下全面贯彻党的十八大和十八届三中、四中、五中全会精神，深入学习贯彻习近平总书记系列重要讲话，协调推进“五位一体”总体布局和“四个全面”战略布局，紧盯脱贫攻坚、推进全面建成小康社会、建设幸福美好新武威奋斗目标，坚持以党建统揽全局、用发展凝聚人心，无中生有抓项目、有中生优增效益，严格标准选干部、不拘一格用人才的理念，竭力实施工业强市、城乡融合、生态立市战略，推动科学发展、转型升级、富民强市，完成了“十二五”规划目标任务，开创了武威经济社会发展新局面，为“十三五”发展奠定了坚实基础。

——综合实力显著增强，发展步入新阶段。2015年生产总值416.19亿元，是2010年的1.8倍，年均增长11.7%，在全省市州的位次由2010年的第7位进到第6位。累计完成全社会固定资产投资3162.8亿元、年均增长34.3%，其中规模以上2534.1亿元、年均增长29.2%；2015年全社会固定资产投资827.64亿元、是2010年的4.4倍，其中规模以上620.1亿元、是2010年的3.6倍。大口径财政收入43.45亿元，是2010年的3.4倍；一般公共预算收入26.84亿元，是2010年的4.2倍，年均增长33.1%，在全省市州的位次由2010年的第12位跨越到第5位；一般公共预算支出171.86亿元，是2010年的2.3倍，年均增长17.9%。招商引资到位资金累计达到2826亿元，是“十一五”时期的27.8倍，年均增长85.4%。金融机构存贷款余额分别达到816.09亿元、696.2亿元，是2010年的2.5倍、4.3倍，年均增长20.3%、34%。

——产业结构优化升级，经济转型步伐加快。三次产业结构由2010年的26.4∶40∶33.6调整为24∶36.6∶39.4，第三产业比重提高5.8个百分点。新型工业体系初步建立，五年累计完成工业投资1135.7亿元，是“十一五”时期的6.6倍，年均增长26.6%。着力培育产业链经济，打造风光电装备制造、碳化硅新材料、生物制药、农产品精深加工、液体经济等产业联盟和产业集群，一批重大骨干工业项目建成。风光电装机规模累计达到300万千瓦。农业主体生产模式基本建成，五年新增设施农牧业67.82万亩，累计达到85万亩，户均2.5亩；新增特色林果基地136.2万亩，累计达到155.66万亩，人均1亩。农产品、特色林果出口基地备案面积分别达到50.24万亩和7.2万亩，设施农产品外销量占总产量的70%以上。甜高粱种植面积扩大到27.3万亩，实施配套加工项目16个。农业增加值99.79亿元，列全省市州第1位。现代服务业快速发展，万嘉国际广场、黄羊公铁联运物流中心、昊天农产品交易市场暨仓储物流中心建成运营，红星时代广场加快建设，市电子商务创业孵化基地和创业实训基地、民勤县电子商务创业孵化园建成投运。华夏文明传承创新区武威项目区加快建设，长城集团武威丝绸之路文化遗产博览城、莫高•中国葡萄酒城等文化产业项目加快推进。我市被命名为“中国葡萄酒城”，连续3年成功举办“中国•河西走廊有机葡萄美酒节”。五年累计完成专利申请2292件，是“十一五”时期的8.1倍；技术市场交易额8.45亿元，是2010年的2.7倍。

——脱贫攻坚扎实推进，贫困面大幅下降。深入推进精准扶贫精准脱贫、“1236”扶贫攻坚行动、“双联富民”行动深度融合，走出了“建棚子、栽林子、抓票子、盖房子、过上好日子”的扶贫开发新路子。2015年，实施“1+17+2”精准扶贫精准脱贫规划方案，建档立卡贫困人口脱贫12.3万人，民勤县整体脱贫。“十二五”时期减贫42.53万人，贫困人口减少到13.54万人，贫困面由35.44%下降到9.1%。科学布局人、水、土资源，大力实施“下山入川”生态移民工程，建成古浪黄花滩、天祝南阳山片、凉州邓马营湖等生态移民示范基地，五年规划7.2万人移民目标超额完成，共搬迁8.43万人。

——交通水利电力建设成效显著，基础设施明显改善。永古、营双、金武高速公路和金色大道、荣生大道、天颐大道、金荣沙漠生态公路、天互二级公路建成通车。干武铁路增建二线和北仙高速、民红高速、天景二级公路进展顺利。新增高等级公路642.6公里，其中高速公路337公里，新建、改建农村公路5875公里，公路总里程达到12317公里，路网密度达到37.1公里/百平方公里。市综合客运枢纽中心建成投运。温德克飞机整机制造和武威机场前期工作扎实推进。建成古浪黄花滩水利骨干工程、天祝南阳山片“下山入川”生态移民小康供水工程、景电二期民调渠延伸和石门河调蓄引水、杂木河毛藏寺水库等重点水利工程。五年累计解决80.15万农村人口、15.46万农村师生的饮水安全问题。新增330千伏变电站3座，新建、扩建110千伏变电站8座，电网供电能力显著提升。

——城乡融合深入推进，新型城镇化提速发展。坚持以城乡融合发展理念统领全市城乡规划，以城乡融合发展核心区建设带动城乡一体化发展。以金色大道为中心轴，优化区域生产力布局，提高公共服务设施共享程度。凉古核心区和民勤、天祝县域核心区综合承载、聚集辐射带动能力显著增强，重点小城镇建设步伐加快，城乡一体化发展新格局初步显现。金色大道城区连接线向南延伸段道路建成通车，市污水处理厂污水再生利用项目建成试运行，天马湖景观治理工程基本建成，滨河公园、滨河东路、城区天然气综合利用等项目加快推进，世行贷款城乡融合发展核心区基础设施项目启动建设，德促贷款城区集中供热项目加快实施，城市功能不断完善。城镇建成区面积达到101.87平方公里。民勤县和凉州区黄羊镇、古浪县黄羊川镇新型城镇化试点有序推进。创建“千村美丽”示范村省级15个、县区级41个，“万村整洁”村319个。常住人口城镇化率由2010年的27.56%提高到35.92%。

——甘肃（武威）国际陆港启动建设，外向型经济起步发展。甘肃首个海关特殊监管区——武威保税物流中心封关运行，中欧国际货运班列“天马号”常态化运营，累计发运33列，货运总量4.68万吨，货运总值1.17亿美元，出口退税、进口保税等口岸功能逐步发挥。武威进境木材检验检疫集中监管区和进口肉类指定查验场获批建设。2015年实现外贸进出口总额6.35亿元，增长162.02%。甘肃路斯宠物食品、甘肃水投陆港中心区水务一体化等项目开工建设，红星美凯龙国际家居产业园和专属保税物流仓库等项目前期工作加快推进。

——石羊河流域重点治理目标提前实现，生态环境持续好转。石羊河流域重点治理任务全面完成，建设工程通过省上竣工验收。蔡旗断面过水量和民勤盆地地下水开采量两大约束性指标、生态治理目标分别提前8年、6年实现，干涸半个世纪之久的青土湖形成水面22.36平方公里，地下水埋深由“十一五”末的3.78米升至3.14米，形成旱区湿地约106平方公里。祁连山水源涵养林、冰川、高山湿地和旱区湿地加快恢复，湿地总面积达到156.3万亩，列全省市州第4位。五年累计完成人工造林251.65万亩、封育180.34万亩，森林覆盖率由2010年的12.1%提高到19.7%。市级财政筹措专项资金1.7亿元建设千里沙漠大林带。严格落实“五禁”决定。深入开展环保大检查，解决了一批违规违法问题。全力打好凉州大气污染防治攻坚战，扬尘、烟尘、尾气和油气综合治理成效明显，2015年实现了空气质量优良天数、PM10年均浓度目标双达标。大力发展循环经济，节能减排目标全面完成。我市被命名为全国节水型社会建设示范区和防沙治沙综合示范区，民勤县被列为首批国家生态保护与建设示范区、高效节水灌溉示范县。

——社会事业协调发展，人民生活水平显著提高。2015年城乡居民人均可支配收入分别达到21702元、9101元，是2010年的1.9倍、2倍，年均增长13.4%、14.9%。城乡居民住房条件明显改善，五年累计建成保障性住房128977套、棚户区改造23138户、农村危房改造78339套，合计占全市总户数的40%，新建新型农村社区126个。城镇累计新增就业13.66万人。社会保障水平稳步提高，城镇居民基本医疗保险制度全面实施，新型农村社会养老制度实现全覆盖，城乡居民养老保险参保率、新农合参合率逐年提高，城乡居民大病医疗保险工作全面启动，企业退休人员基本养老金、失业保险金、工伤人员伤残津贴标准提高10%，连续5年提高城乡低保、农村五保供养标准。义务教育步入均衡发展新阶段，学前教育和高中教育加快普及。实施“全面改薄”项目，D级危房全面消除。职业教育稳步发展，组建武威职业教育集团，武威职业学院国家骨干高职院校建设项目通过评估，一期扩建工程加快推进。整合市、区中医医院，组建武威职业学院直属附属医院。创办兰州交通大学新能源与动力工程学院、甘肃威龙葡萄酒业专修学院。医疗卫生服务体系不断完善，县级公立医院综合改革全面展开，中国首台自主研发的医用重离子加速器建成并成功出束，市人民医院整体搬迁运行，市中医医院综合服务楼建成投用。恶性肿瘤高发区防控模式示范项目全面完成，乙肝防治示范区建设项目持续推进。市博物馆、体育馆、影剧院主体建成。五年累计建成乡村综合性文化服务中心（乡村舞台）730个、乡镇及社区体育健身中心43个。群众就业、就学、就医、出行、饮水等状况明显改善，城乡基本公共服务水平不断提升。

——改革全面深化，发展动力不断增强。大力简政放权，承接、取消、下放、调整了一批行政审批事项。公共资源交易中心和“12345”便民服务中心建成投运。积极开展“三张清单一张网”工作，市县区政府部门权责清单全部公开发布。将凉州城区土地、建设管理权下放凉州区，工商行政管理、公安交警管理体制改革任务全面完成。全面启动“三证合一”和“一照一码”，2015年新增市场主体18567户，非公经济增加值占生产总值的比重达到63.5%。改革创新融资方式，累计发放“妇小贷”、“双联贷”、“双业贷”和精准扶贫贷款、易地扶贫搬迁贷款等五项财政惠农贷款315亿元，农户户均9万元，覆盖面80%，各级财政贴息25亿元。整合改制经济发展集团，盘活交通、水务、城建等优质国有资产，着力打造融资平台。119家企业在甘肃股权交易中心挂牌，1家企业在“新三板”挂牌融资。组建武威农商银行、民勤农商银行，设立甘肃银行、兰州银行、交通银行分支机构，基本实现农村金融服务全覆盖。成功发行企业债券和私募债券，设立工业发展财政引导基金和现代农业产业创投基金各3亿元。在全省率先开展农村产权确权颁证和抵押贷款工作，设施农牧业产权登记颁证13.36万件，特色林果确权颁证45.41万亩。我市连续3年获得“省长金融奖”。大力培育新型农业生产经营主体，成立市农民专业合作社联合会，农民专业合作社达到7307家。土地流转面积88.4万亩，土地规模经营面积23.2万亩，治沙生态林承包经营面积180.9万亩。推进省级创新型试点城市和甘肃武威国家农业科技园区建设。坚持管好用活土地，土地资产收购、储备、开发、出让制度进一步完善。加大人才引进力度，累计引进各类人才1262人，其中博士25名、硕士362名。

——政府自身建设加强，依法行政能力不断提高。坚决贯彻中央八项规定和省委、市委“双十条”规定，扎实开展党的群众路线教育实践活动和“三严三实”专题教育，整治贪假懒庸、为官不为，“四风”积弊和不严不实问题逐步解决。严格落实党风廉政建设“4883”主体责任体系，扎实开展“四述”和廉政约谈，层层传导压力，干部作风逐步好转。坚持依法行政，2015年组织开展“依法建设、规范运行、敢于担当、健康发展”专项检查，围绕环境保护、土地利用、安全生产等11个方面64类问题，对378户重点企业、973个固定资产投资500万元以上项目开展集中检查，督促整改存在问题。集中开展依法打击玉米非法制种专项行动。建立“欠薪黑名单”制度，依法查处克扣、拖欠农民工工资案件。严格落实安全生产责任制，加强安全生产基层基础工作，开展安全生产大检查，安全生产形势总体稳定。全面贯彻落实党的民族政策和宗教工作方针，有力促进了民族团结、宗教和顺。军政军民关系更加融洽，国防动员和“双拥”工作进一步加强，我市再次被评为省“双拥模范城”。统计、气象、人防、消防、邮政、老龄、外事侨务、防震减灾、档案管理、地方志、妇女儿童、残疾人和慈善事业等工作取得新的成绩。

2015年，我们认真落实国家、省上一系列政策措施和市委决策部署，积极适应经济发展新常态，努力克服经济下行压力持续加大等不利因素，全力推动经济社会转型升级发展，基本完成了市三届人大四次会议确定的主要目标任务。全市呈现出经济运行稳中有升、生态环境明显好转、人民生活持续改善、改革开放稳步推进、依法治市全面加强、社会大局和谐稳定的良好局面。生产总值增长8.7%，规模以上工业增加值增长4.1%。全社会固定资产投资增长15.02%，其中规模以上固定资产投资增长12.8%。大口径财政收入增长18.44%，一般公共预算收入增长21.02%，一般公共预算支出增长14%。城镇居民人均可支配收入增长9.2%，农村居民人均可支配收入增长11.6%。居民消费价格指数101.5%。全市金融机构本外币存款余额增长15.83%，贷款余额增长31.87%。

各位代表，项目工程实物实体和法定数据显示，“十二五”时期是武威历史上项目建设最多、投资规模最大、发展速度最快、综合效益最好的时期，也是基础设施和生态环境改善最明显、城乡面貌变化最显著、社会治理成效最突出、人民群众得到实惠最多的时期。这是省委、省政府和市委正确领导的结果，是市人大、市政协和社会各界监督支持的结果，是全市各族人民团结一心、艰苦奋斗的结果。我代表市人民政府，向奋战在各条战线的劳动者，向人大代表、政协委员，向各民主党派、工商联、无党派、人民团体和社会各界人士、离退休老同志，向驻武部队、武警官兵和中央、省属在武各单位以及所有关心支持武威改革发展稳定的同志们、朋友们，表示崇高的敬意和衷心的感谢！

回顾过去的工作，我们也清醒认识到，政府工作与人民群众的期待还有差距，经济社会发展还存在不少困难和问题。发展不足、经济总量小、人均水平低、小康实现程度低等问题还没有从根本上解决；市场主体总量较少，缺少竞争实力强的骨干企业、能够主导产业的大项目、好项目和品牌产品；城乡基础设施依然滞后，城镇化水平和公共服务均等化程度相对较低，公共服务供给主要依靠国家政策支持，自主保障能力弱；生态脆弱的状况没有根本改变，生态环境刚性约束趋紧，节水、治沙、造林任务重，治理大气污染、改善环境质量仍需不懈努力；剩余贫困人口贫困程度深、脱贫难度大，实现全面脱贫任务十分艰巨紧迫；一些干部思维方式和工作作风还不适应新形势新任务的要求，责任意识、服务意识、担当意识不强，苦干实干劲头不足，庸政懒政怠政、伪善伪廉伪勤等问题时有抬头。对这些问题，我们将高度重视，切实加以改进和解决。

“十三五”经济社会发展的指导思想和目标任务

“十三五”时期是全面建成小康社会、实现第一个百年奋斗目标的决胜阶段，也是经济社会转型升级、建设幸福美好新武威的关键阶段。根据《中共武威市委关于制定国民经济和社会发展第十三个五年规划的建议》，紧密结合实际，在广泛征求意见、集中各方智慧、充分研究论证的基础上，编制了《武威市国民经济和社会发展第十三个五年规划纲要（草案）》，分析了“十三五”时期发展的基础和环境，提出了经济社会发展的指导思想、主要目标、重点任务及保障措施。

全市“十三五”经济社会发展的指导思想是：高举中国特色社会主义伟大旗帜，全面贯彻党的十八大和十八届三中、四中、五中全会精神，深入学习贯彻习近平总书记系列重要讲话，认真落实省第十二次党代会和省委十二届十四次全委会议精神，按照“五位一体”总体布局和“四个全面”战略布局，坚决全面从严治党，切实加强党的领导。坚持以人民为中心的发展思想，坚持发展是第一要务，坚持以经济建设为中心不动摇，坚持创新发展、协调发展、绿色发展、开放发展、共享发展理念。坚持以党建统揽全局、用发展凝聚人心，无中生有抓项目、有中生优增效益，严格标准选干部、不拘一格用人才，决战决胜脱贫攻坚，大力推进创新驱动，实施开放外向、工业强市、城乡融合、生态立市战略。坚持以提高发展质量和效益为中心，以转型升级为主线，强化发展基础，推动经济总量上台阶、质量效益上水平，壮大经济实力。坚持经济发展与环境、资源、安全相协调，坚持经济建设与社会事业发展同步、与持续增加城乡居民收入并举，着力构建适应新常态的体制机制和发展方式，统筹推进经济、政治、文化、社会、生态文明建设和党的建设，加快建设经济富强、人民幸福、风清气正、环境优美的新武威，与全国全省一道全面建成小康社会。

主要目标：与全省一道全面建成小康社会并达到较高层次。

一、经济保持中高速增长。在提高发展平衡性、包容性、可持续性的基础上，推动经济总量上台阶、质量效益上水平。生产总值年均增长9%，高于全省平均水平，到2017年比2010年翻一番，到2020年突破600亿元。固定资产投资年均增长12%，投资方向、结构、效益得到优化和提高。一般公共预算收入年均增长11%，收入结构不断优化，财政保障能力进一步增强。社会消费品零售总额年均增长11%，消费对经济增长的贡献明显加大。

二、产业迈向中高端水平。发展壮大支柱产业，培育形成新产业新业态，推动产业集群发展，服务业比重进一步提高，多元支撑、竞争力强的现代产业体系基本形成，现代农业、新型工业、现代服务业和外向型经济协同发展。科技创新能力不断增强，研究与试验发展经费投入强度力争达到2%，科技进步贡献率达到55%以上。

三、人民生活水平普遍提高。现行标准下贫困人口2017年全部脱贫，贫困县全部摘帽。城乡居民人均可支配收入年均增长10%，2016年比2010年翻一番，到2020年分别达到34950元、14650元，分别比2010年增长2倍、2.2倍。实现更加充分的就业，城镇累计新增就业14万人，城镇登记失业率控制在4%以内。健康环境进一步优化，基本公共服务均等化水平稳步提高，社会保障体系覆盖城乡居民。

四、公民素质和社会文明程度全面提升。中国梦和社会主义核心价值观深入人心，公民思想道德素质、法律素质、科学文化素质、健康素质明显提高，城乡文明程度全面提升。到2020年，普及学前三年教育，九年义务教育巩固率保持在99.5%以上，高中阶段毛入学率达到95%以上；公共文化服务体系基本建成，文化产业增加值占生产总值比重达到5%，特色文化市建设取得重大进展。

五、城乡融合发展取得新成效。城乡空间结构和生产力布局更趋优化，城乡融合发展核心区集聚和辐射带动作用进一步增强，新型城镇化建设步伐加快，常住人口城镇化率达到50%以上，城镇化水平高于全省平均水平。城乡一体化发展取得新成效，推动形成产业发展城乡联动、基础设施城乡配套、公共服务城乡协调、社会保障城乡覆盖的新格局。

六、生态环境质量明显改善。节水、治沙、造林、防污取得新成效，石羊河流域重点治理成果不断巩固提高，节水型社会全面建成，主体功能区布局不断优化，国家生态安全屏障综合试验区和全国生态文明示范区建设取得明显进展。森林覆盖率达到22%以上。循环经济水平进一步提升，节能减排取得明显成效，单位地区生产总值能耗、万元地区生产总值用水量、主要污染物排放总量、大气污染防治指标控制在省政府下达目标以内。地表水监测断面水质、县乡集中式饮用水源地水质达标率达到规划目标，城市生活污水处理率达到95%、生活垃圾无害化处理率达到99%以上。

七、改革开放实现重大突破。着力加强供给侧结构性改革，重点领域和关键环节改革取得决定性成果。政府职能转变成效明显，投融资体制不断创新，非公经济占生产总值比重达到70%左右，质量效益明显提升。持续营造“能赚钱、不受气、条件好”的投资环境，招商引资水平显著提高。甘肃（武威）国际陆港建成，对外开放深度和广度不断拓展，外向型经济迈上新台阶，外贸进出口总额达到10亿美元以上。

八、治理体系和治理能力现代化取得新进展。依法治市全面推进，民主法制更加健全，法治政府基本建成，司法公信力明显提高，全社会法治意识普遍增强，政府建设科学化水平显著提高。安全发展理念牢固树立，安全生产基础切实加强，安全生产责任制全面落实。法治武威、平安武威、和谐武威建设取得新进步，人民权益得到切实保障，社会大局保持和谐稳定，民族团结、宗教和顺局面不断巩固。

《武威市国民经济和社会发展第十三个五年规划纲要（草案）》经大会审议通过后，市政府将组织有关部门以《纲要》为依据，编制若干专项规划，细化分解和落实《纲要》提出的各项任务。

2016年主要工作

2016年是实施“十三五”规划的开局之年，也是全面建成小康社会决胜阶段的开局之年。根据中央、省上总体要求和市委三届十次全委会议、市委经济工作暨扶贫开发工作会议部署，2016年全市经济社会发展主要预期目标是：生产总值增长9%，工业增加值增长9.2%。全社会固定资产投资增长13%，其中规模以上增长10%。一般公共预算收入增长15%。社会消费品零售总额增长10%。进出口总额增长20%。城镇居民人均可支配收入增长9%，农村居民人均可支配收入增长11%。居民消费价格指数涨幅控制在3%以内。城镇化率提高2.9个百分点。单位生产总值能耗和主要污染物排放量完成省政府下达目标。

围绕实现上述目标，重点抓好十个方面的工作。

一、全面推进精准扶贫，坚决打好脱贫攻坚战。扎实推进“双联富民”行动和精准扶贫深度融合，全力实施“1+17+2”精准扶贫精准脱贫规划方案，全面推行“853”挂图作业，确保全市减贫104个贫困村、27692户、10.71万人，凉州区整体脱贫。坚持精准扶贫与生态保护相结合，加快“下山入川”易地扶贫搬迁，确保完成4万人以上搬迁任务，其中建档立卡贫困户3783户、15423人。加快建设古浪绿洲移民基地小城镇、天祝生态移民藏族村庄、凉州区邓马营湖等移民安置点，对“下山入川”移民区按小城镇标准规划建设，对留居山区的农户统一规划、收缩居住。创新和强化移民区社会管理服务，保障移民群众乐业安居。持续推进贫困片区主体生产模式提质增效，实现富民产业全覆盖，确保移民搬得下、稳得住、快致富。扶持发展农产品加工业和流通业，培育发展富民沙产业，开展电商扶贫，发展乡村旅游和休闲农业。加快实施建制村通畅工程、“千村美丽”示范村村组道路硬化工程和农村公路安全生命防护工程，年内全面实现所有建制村通沥青（水泥）路、通班车目标。推进精准扶贫饮水安全项目，解决23个贫困村、5488户、2.52万贫困人口饮水安全不稳定问题。实施农村危房精准到村到户改造工程，完成建档立卡贫困户年度危房改造任务。推动贫困片带光缆入乡、入村、入户，实现贫困村宽带网络全覆盖。加大教育扶贫力度，推进学前教育资源向贫困村延伸，改善贫困村义务教育办学条件。加大贫困乡村文化基础设施建设力度，建成“乡村舞台”104个。健全卫生计生服务体系，实现贫困村标准化村卫生室全覆盖。加快实施精准脱贫恶性肿瘤防控工程，解决因病致贫返贫问题。完善财政扶贫投入稳定增长机制，当年财政收入增量天祝县和古浪县按20%以上、凉州区和民勤县按15%以上增列专项扶贫预算。加强扶贫资金监管，认真开展“两查两保”专项行动，严肃查处挤占挪用、层层截留、虚报冒领、挥霍浪费扶贫资金等行为。完善精准扶贫考评体系，落实动态考核机制。

二、持续加快项目建设和招商引资，增强经济发展后劲。坚持把招商引资抓项目、增加投资扩总量作为推动经济发展的主动力，加大力度，积极作为，持续推进。力争全年实施500万元以上项目600项以上，确保在建项目规模保持在1000亿元以上。创新招商引资方式，集中力量招大引强，力争招商引资到位资金达到1000亿元。加强基础设施建设，干武铁路增建二线、民红高速公路、天景二级公路、千里沙漠大林带治沙道路年内建成通车。北仙高速公路建成武威至民勤段和绕城高速，开工武威至仙米寺段。力争建设兰张铁路三四线。立项建设古浪大靖至干城、凉州区至古浪公路、民勤大滩至南湖公路、古浪干城至西沟公路、凉古公路至沙漠公园公路、金色大道至白塔寺景区公路等项目。建成天祝二道墩注入式调蓄水库并投入运行，基本建成甘肃（武威）国际陆港中心区、民勤红沙岗工业园区供排水一体化工程等项目，争取建成红崖山水库加高扩建主体工程，实施一批区域供水、高效节水、农田水利等基础设施建设工程。开展引黄济石沙漠治理暨精准扶贫移民供水工程前期工作。加快推进甘电投武威热电联产、亿利资源集团武威工业园区微煤雾化热电联产项目，建成杂木河神树水电站。

三、举全市之力建设甘肃（武威）国际陆港，全面提升外向型经济发展水平。实施《甘肃（武威）国际陆港发展战略规划》，加快建设以武威保税物流中心为核心的陆港中心区，突出保税、物流、出口、对外加工等核心功能，全力打造功能完备、要素聚集、运行高效、具有国际影响力的甘肃（武威）国际陆港，提升丝绸之路经济带武威黄金节点战略地位和作用。甘肃路斯1.8万吨宠物食品生产项目建成投产，开工建设国际家居产业园等项目和武威保税物流中心铁路专用线及铁路集装箱作业区，加快推进武威机场、武威铁路物流中心前期工作。建成武威进境木材检验检疫集中监管区、武威进口肉类指定查验场，着力打造木材产业集聚区和进口木材集散中心、内陆地区进口肉类产品集散地。申报设立武威铁路一类口岸、进口粮食等指定口岸。加强与丝绸之路经济带沿线国家和地区的合作交流，组织企业参加国内外知名博览会和专业博览会，引导企业开拓国际国内市场。扩大产品出口，加快培育出口企业，打造向西开放的出口产品加工基地，创立武威品牌，扩大装备制造、新材料、葡萄酒等工业产品市场占有率。高标准建设农产品出口基地，重点培育蔬菜、果品、肉羊、奶制品和皮毛等出口产品，出口农产品生产基地达到70万亩，辐射带动周边区域面积120万亩。积极搭建产品出口服务平台，吸引进出口产品和加工贸易企业在武威集结，努力把武威打造成全省外向型经济发展龙头。

四、加快建设新型工业体系，推动工业转型升级。认真落实《中国制造2025武威实施方案》，重点推进高端装备制造、新材料、生物技术、信息技术等高新技术产业发展，新开工千万元以上工业项目110项，建成120项。开工建设医用重离子加速器装备、出口实木家具等制造项目，积极推进通航飞机制造项目前期工作，争取发展新能源汽车制造产业。抓好2兆瓦电励磁低速同步发电机和小容量混凝土搅拌运输车研制项目。加快天祝松山滩百万千瓦级风电基地建设，扎实做好金武千万千瓦级风光互补发电基地前期工作。加快推进遍净集团医药中间体项目，建设精细化工产业链，打造民勤红沙岗精细化工园区。加快甜高粱深加工项目建设，引进实施生物质能、聚乳酸及下游应用产品、饲料加工等项目，推动甜高粱战略性新兴产业集聚发展。建立碳化硅新材料产业技术联盟，积极申报“国家火炬武威市碳化硅新材料特色产业基地”。提高节水灌溉、管道等先进装备制造水平。实施“互联网+”行动，推进大数据、物联网等信息技术在制造业全产业链中集成运用。支持青啤、达利、鑫淼、荣宝、凯帝斯、华一、中大铝材等骨干企业达产达标，推进多功能立体固沙车、甜高粱收割机、荣华乳品、中钢玉通等重点项目建成投产。深入开展“两年百户企业达标入规”行动，新增规上企业62户。用高新技术和先进适用技术改造提升传统产业，引导和支持企业与省内外高校、科研院所开展合作，加快科技成果转化应用，促进产业转型升级。新认定省、市级企业技术中心（工程技术研究中心）10家以上，推广新技术15项以上，开发市级以上新产品50项。加强工业运行调度，落实工业项目奖补、贴息等扶持政策，支持企业完善环保等设施，引导企业完善管理制度，强化煤电油运等要素保障，深入推进重点项目互保共建和工业企业互为市场工作，确保工业经济稳定增长。加快省级循环经济示范试点园区和企业建设，组织实施一批工业循环经济重点支撑项目，落实工业节能降耗目标，确保全面完成节能减排各项目标任务。

五、持续推进主体生产模式提质增效，不断增加农民收入。围绕实现设施农牧业棚均收入2万元、特色林果亩均收入5000元目标，大力推广“下双模式”，加强标准化建设、规范化管理、集约化经营，加大新品种和先进适用技术引进推广力度，鼓励支持多元化特色种养，积极创建省部级蔬菜标准园、畜禽养殖标准化示范场，加强动植物疫病防控，提升主体生产模式综合效益。加快建立质量安全追溯体系，加强农产品质量安全监管。加大“三品一标”认证力度，培育一批农产品和企业品牌。强力推进“贮藏加工+运输销售”现代营销模式，发展订单式设施农业，加快农产品冷链仓储和流通设施建设，强化农村专业合作社和农业产业化龙头企业带动作用，扩大特色农产品市场占有份额。加快甜高粱种植基地建设，力争完成40万亩种植任务。推广高效农田节水技术250万亩、旱作农业70万亩。

六、大力发展现代服务业，培育新的经济增长点。加快发展现代物流业，提升万嘉国际广场、黄羊众远公铁物流中心、武威昊天农产品交易市场运营管理水平，加快红星时代广场、盛朝金融大厦等商贸物流项目建设，开工建设武威万达广场，加快发展大型商场、连锁超市，建设和改造生产资料市场、消费品市场、商品配送中心，构建现代物流配送体系和城乡商业新网络。加快发展电子商务，建立县乡村三级电子商务服务体系，建设民勤县国家级电子商务进农村综合示范县和凉州区省级电子商务示范县（区），深入推进“武威特产网上行”促销工程，培育网络品牌。积极发展服务外包、工业设计和研发设计、文化创意、节能环保、养老服务、健康服务等新兴服务业。加快长城集团武威丝绸之路文化遗产博览城、莫高•中国葡萄酒城文化产业园、雷台文化产业园、塔儿湾冰雪嘉年华二期工程、松山古城及赛马场滑雪场、民勤朗福生态文化旅游景区等项目建设。继续办好“中国•河西走廊有机葡萄美酒节”。全面实施金色大道•马踏飞燕大景区规划和天祝民族风情山水生态游规划，积极稳妥推进大景区管理体制改革，改善景区基础设施和服务设施，提升服务管理层次。实现旅游接待人数959.49万人次，旅游综合收入49.54亿元、增长24.2%。

七、深入实施城乡融合战略，推进新型城镇化进程。加快建设城乡融合发展核心区，推动城乡一体化发展。按照城乡融合和“多规合一”要求，修编实施武威市城市总体规划、土地利用总体规划、历史文化名城保护规划、新型城镇化规划，完善县城、园区、小城镇、新型农村社区和村庄规划，编制综合交通、市政基础设施等专项规划，建立定位清晰、统一衔接、功能互补的规划体系。维护规划严肃性，严把城市主要节点和单体建筑规划设计，加强建筑物色谱管理，新建项目和政府投资项目要严格执行城市建筑物色谱管理规定。统筹推进城乡交通、水利、通讯、电力、环保、信息化、停车场、新能源汽车充电桩（站）等基础设施建设。积极推进凉州城区地下综合管廊建设，加快实施德促贷款城区集中供热、城市供水、污水管网改造和建设项目。全面建成天马湖景观治理工程、滨河公园、滨河东路，整合天马湖以东土地资源，发展商务商贸、休闲旅游、会展等服务产业，打造东片商贸居住中心。加快海藏湖生态治理工程。加强历史文化名城保护，开工建设文庙、古钟楼、罗什寺等历史文化街区。加快万嘉汇•台湾士林不夜城等城市综合体项目建设，争取开工建设天马中心项目。持续改善居民住房条件，加大棚户区改造力度，提高货币化安置比例，配套完善保障性安居工程基础设施，加快已建成保障房分配入住。加强城市综合执法，提升城市治理水平。加快民勤、古浪、天祝县城和重点小城镇建设，全面推进新型城镇化试点建设，加快移民点、新型农村社区规划建设，新建、续建新型农村社区52个。规范新型农村社区管理，集中整治村容村貌。积极创建省级“千村美丽”示范村和“万村整洁”示范村。

八、全力实施生态立市战略，构筑生态安全屏障。统筹抓好“节水、治沙、造林、防污”四个重点，加快建设全省生态环境保护示范区、全国防沙治沙综合示范区和生态文明示范区。强化水资源调度管理，确保平水年份蔡旗断面过水量达到2.9亿立方米以上、民勤盆地地下水开采量控制在0.86亿立方米以内，全市用水总量控制在16.46亿立方米以内，地下水开采量控制在4.84亿立方米以内。大力推广综合节水技术，加快推进河西走廊国家级高效节水灌溉示范区项目建设。完成石羊河流域重点治理规划总体评估，积极谋划石羊河流域后续治理项目。加快推进三北防护林、新一轮退耕还林还草、沙化土地封禁保护、千里沙漠大林带工程、濒危野生动物民勤青土湖试验性放养基地等重点生态建设项目，争取实施祁连山生态保护与建设综合治理规划，抓好“下山入川”移民迁出区、矿区塌陷区生态恢复治理，依法依规清理整治祁连山国家级自然保护区内建设项目，加强民勤石羊河国家湿地公园和青土湖、黄案滩等荒漠湿地保护建设。完成人工造林35万亩，封山（沙）育林（草）36万亩。大力开展工业治沙、生物治沙、机械治沙、产业治沙和治沙新材料、新技术、新品种的试验示范，加快发展沙漠特色养殖和林果、药材种植产业，推进治沙林承包治理经营，促进沙区生态治理。加强环境保护，落实环保责任，严格执行“五禁”决定，实施大气、水、土壤污染防治行动计划，确保完成总量减排目标。继续打好凉州大气污染防治攻坚战，落实治污、控煤、抑尘、管车、禁燃等措施，努力完成空气质量优良天数、PM10和PM2.5年均浓度目标任务。天祝、古浪、民勤县同步推进扬尘、烟尘、油气和尾气综合整治。实施工业点源、农业面源和城乡生产生活污染治理，严禁无证排污、超标准和超总量排污，实现污染治理全防全控。

九、持续改善民生，增强人民群众的获得感。贯彻共享发展理念，协调发展社会事业，提高公共服务水平。落实更加积极的就业创业政策，城镇新增就业2.2万人以上，应届高校毕业生就业率保持在85%以上，城镇登记失业率控制在4%以内。认真做好机关事业单位养老保险工作。加大城乡居民养老保险和被征地农民社会保障政策落实力度。推进城乡居民基本医疗保险制度整合，全面实施城乡居民大病保险。实行城乡孤儿基本生活补助统一标准。扩大社会保障覆盖面，推进社会保险“五险合一”。加强养老基础设施建设，提高养老水平。重视解决城市困难群体的问题。加大救助供养力度，对特困人员应救尽救、应养尽养。加快学前教育发展，新建、改建村级幼儿园74所。推进“全面改薄”项目，促进义务教育均衡发展，确保凉州区、天祝县通过国家义务教育发展基本均衡县评估认定。加强师资队伍建设，提升基础教育质量，普及高中阶段教育。实施武威一中迁建提升工程，积极开展省级示范性普通高中创建工作。加大对兰州交通大学新能源与动力工程学院的支持力度，落实支持武威职业学院发展的政策措施，提升办学水平。全面推进县级公立医院综合改革，加强县级医院重点专科项目建设，完成27个标准化村卫生室建设。积极创建全省中医药工作先进市。全面落实二孩政策，促进人口均衡发展。加快推进城市社区和农村综合性文化服务中心（乡村舞台）建设，建设乡镇及社区体育健身中心7个、村级体育场120个。加强安全生产，严格落实政府领导责任、部门监管责任、企业主体责任，深入开展安全生产大检查，加大执法监管力度，实行安全生产巡查常态化，坚决防止重特大事故发生，保持安全生产形势稳定。强化食品药品安全监管，确保人民群众饮食用药安全。健全完善社会治安防控体系，深化平安武威建设，切实保障公共安全。深入开展“防风险、保安全”风险隐患排查化解专项行动，健全和落实长效工作机制。深入开展民族团结进步创建活动，依法加强民族宗教事务管理，确保社会大局和谐稳定。

围绕解决群众最关心最直接最现实的利益问题，全力办好十件实事：1、完成建制村通沥青（水泥）路590公里，实现全市所有建制村通沥青（水泥）路。2、实施5.5万农村人口饮水工程。3、实施精准脱贫恶性肿瘤防控工程，免费开展胃癌防控和乙肝、妇女“两癌”普查10万人。4、实施农村危房改造10020户、新房建设12980户。5、实施棚户区改造9392户。6、新建改建扩建福利机构，对无劳动能力、无生活来源、无法定抚养人或赡养人的“三无”残疾人全部实现集中供养。7、提高城市低保标准、农村五保供养补助标准和农村一、二类低保标准，实现与扶贫脱贫线“两线合一”。8、对全市学前教育幼儿免（补助）保教费，对建档立卡贫困家庭普通高中学生免（补助）学杂费和书本费。9、实现农村宽带服务行政村全覆盖。10、创建200所“宽带网络校校通”和“优质资源班班通”中小学。

十、落实全面深化改革政策措施，释放发展活力。加强供给侧结构性改革，抓好降低企业成本、消化房地产库存等工作，落实税收改革措施，取消更多行政管制，解除对人口和劳动力、土地和资源、资本和金融、技术和创新、制度和管理等的供给抑制，提高供给质量和效率。加快推进大众创业、万众创新。深化农村集体产权制度改革，加快农村房屋、设施农牧业、特色林果业产权和农村承包土地经营权确权颁证进度，全面推进农业水价综合改革，扎实推进集体林权制度配套改革和国有林场改革，完善林地、林木流转机制，全面推行供销合作社综合改革。深化商事制度改革，全面推进“三证合一”和“一照一码”，强化“先照后证”事中事后监管，加快培育一批“专精特新”中小企业。认真落实公平市场准入、公平资源配置、拓展投资领域、鼓励加强自主创新等支持民营经济发展的政策措施，实施市场主体增量提质行动，建立新型政商关系，促进非公有制经济健康发展。深化预算管理制度改革，加大财政资金统筹力度，盘活财政存量资金，调整优化支出结构，强化财政资金监管，集中财力办大事。全面推开“营改增”试点工作，坚持依法征收，做到应收尽收。推进投融资体制改革创新，鼓励和支持社会资本进入基础设施、生态环保、市政公用事业和其他公共服务领域。鼓励企业上市融资或到股权交易市场挂牌融资。发挥财政资金杠杆作用，探索发行项目债券、市政债券，运用资产证券化、设立产业投资基金等方式，提高政府融资能力。启动凉州区全国农村承包土地经营权抵押贷款试点工作。完成古浪县、天祝县农信社改制组建农商银行，加快金融分支机构向新型农村社区、“下山入川”移民点、产业园区延伸。健全完善政银企合作机制，引导金融机构定向培育和扶持中小企业“达标入规”。加强金融生态环境建设，健全完善征信体系。坚持管好用活土地，强化商贸、工业、物流等重点项目用地保障，严格执行耕地占补平衡制度，落实城乡建设用地增减挂钩政策，推进城镇低效、闲置用地再开发利用和工矿废弃地、闲置农村宅基地复用。深化户籍制度改革，全面实行居住证制度，鼓励有能力在城镇稳定就业和生活的农业转移人口举家进城落户。落实户籍变动与土地承包经营权、宅基地使用权、集体收益分配权“三权”脱钩政策，解决好医疗、养老、社保等问题，使进城农民生活有多重保障。持续推进简政放权，放管结合、优化服务，全面实施政府权力清单、责任清单、推行市场准入负面清单制度，加快推进“网上行权”，规范行政权力运行。全面落实公务用车制度改革措施，稳妥推进事业单位改革。严格控制机构编制，完善动态调整机制。

各位代表！实现全面建成小康社会宏伟目标，完成今年经济社会发展目标任务，我们必须按照“为民、务实、清廉”的要求，大力加强政府自身建设。

一是坚持依法行政。带头尊法学法守法用法，运用法治思维和法治方式破解难题、化解矛盾、维护稳定、推进发展。坚持依法决策、依法行政，养成遇事找法、办事依法、解决问题靠法的行为习惯，在法治框架内落实改革发展稳定各项任务。自觉接受人大及其常委会的法律监督、工作监督和政协的民主监督，自觉接受社会舆论和人民群众的监督。加强效能监察，严肃追究滥用权力和失职渎职行为。减少和规范行政审批事项，加快电子政务建设，推进政务公开，确保行政权力在阳光下运行。

二是坚持为民务实。坚持人民主体地位，牢固树立以人民为中心的发展思想，把增进人民福祉、促进人的全面发展作为工作的出发点和落脚点，竭力抓发展，全力惠民生。坚持深入基层，倾听群众呼声，了解群众诉求，真心实意为群众办实事，认真解决征地拆迁、环境保护、劳动争议、涉法涉诉等事关群众切身利益的具体问题，对困难群众给予更多关注、关心和帮助，让广大人民群众共享改革发展成果。

三是坚持提升能力。加强政策理论学习，吸收新信息，研究新情况，掌握新知识，总结新经验，准确把握经济形势和发展规律，突破传统思维束缚，创新工作方式方法，切实解决一些干部无所适从“不会为”、能力不足“不能为”、动力不足“不想为”、担当不足“不敢为”的问题，提高推动科学发展、构建和谐社会、依法行政的能力。

四是坚持改进作风。牢记作风建设永远在路上。深化拓展党的群众路线教育实践活动和“三严三实”专题教育成果。扎实开展“两学一做”学习教育，突出基础在学、关键在做，把学习党章党规与学习习近平总书记系列重要讲话结合起来，增强政治意识、大局意识、核心意识、看齐意识，始终忠诚于党、始终对组织坦诚、始终正确对待权力、始终牢记政治责任。坚持问题导向，持续整治贪假懒庸，着力解决不担当、不作为、慢作为等突出问题。严格执行中央八项规定和省委、市委“双十条”规定，持之以恒反对“四风”。深入开展效能风暴行动，提高行政效能和服务水平。

五是坚持廉政勤政。严格落实“4883”主体责任体系，认真履行党委（党组）集体领导责任、主要负责人第一责任和班子其他成员职责范围内的领导责任，推进主体责任向基层延伸。严格执行《中国共产党廉洁自律准则》和《中国共产党纪律处分条例》，始终把纪律和规矩挺在前面，零容忍、下狠手，严肃查处违法违纪案件，着力解决发生在群众身边的不正之风和腐败问题。强化源头治腐，加强制度刚性约束，严格对财政资金、工程建设招投标、土地使用权出让、产权交易、政府采购等重点领域的监管，加大审计监督和专项巡察力度。政府班子成员要严于律己、以身作则、以上率下，推进干部清正、政府清廉、政治清明，进一步形成凝心聚力抓发展、风清气正干事业的良好政治生态。

各位代表，“十三五”发展的美好蓝图已经绘就，我们责任重大、使命光荣。让我们在省委、省政府和市委的坚强领导下，解放思想，开拓创新，真抓实干，砥砺奋进，为建设幸福美好新武威、实现与全国全省一道全面建成小康社会目标而努力奋斗！
